# Que Peças Faltam no Quebra-Cabeça da Adaptação Cardiovascular ao Ortostatismo?

**DOI:** 10.36660/abc.20230417

**Published:** 2023-07-27

**Authors:** Jorge Elias

**Affiliations:** 1 Vitória Apart Hospital Serviço de Eletrofisiologia Serra ES Brasil Vitória Apart Hospital – Serviço de Eletrofisiologia, Serra, ES – Brasil; 2 Hospital Universitário Cassiano Antonio Moraes Ufes Vitória ES Brasil Hospital Universitário Cassiano Antonio Moraes (Hucam) – Ufes, Vitória, ES – Brasil

**Keywords:** Síncope, Teste de inclinação, Variabilidade R-R, Velocidade da Onda de Pulso, Ortostase

O bipedalismo da espécie humana é um marco evolutivo e tem motivado extensas pesquisas nas áreas fisiológica e antropológica.^1 , 2^

Embora a força gravitacional (+Gz), atuando a uma velocidade média de 9,8 m/s^2^ , crie um gradiente de pressão ao nível do sistema circulatório, o ser humano é capaz de manter uma postura ereta porque a pressão gravitacional é parcialmente neutralizada por mecanismos compensatórios autonômicos e cardiovasculares que impedem a hipotensão e a perda de consciência após a ortostase.^1 - 3^

Nesta edição, Oliveira et al.,^4^ contribuem com mais algumas peças para este complexo quebra-cabeça de adaptação ao estresse gravitacional em crianças e adolescentes.^4^

Recentemente, observou-se uma diminuição do uso do teste de inclinação (TI) na investigação de síncope. Alguns autores chegaram a argumentar que o TI para investigação de síncope deveria ser abolido devido à possibilidade de não estabelecer uma causa explícita de síncope; ter muitos falsos positivos e nunca desempenham um papel na orientação do tratamento.^5^

Embora o TI ao longo do tempo ser utilizado na estratificação diagnóstica mais relacionada a subgrupos específicos de pacientes com história de síncope, persiste como método diagnóstico seguro, de baixo custo, reprodutível e de baixo risco para investigação da resposta ao estresse gravitacional e para estudar a fisiopatologia relacionada à síncope e outras patologias relacionadas ao desajuste ao estresse ortostático.

Essa observação é corroborada pelo presente estudo, onde uma abordagem clínica integrada, utilizando a história clínica, o escore de Calgary e o teste de inclinação, possibilitou o diagnóstico etiológico na grande maioria dos pacientes, evitando assim exames desnecessários.^4^

Alguns aspectos se destacam neste estudo.

O primeiro foi que diversas variáveis clínicas e os escores de Calgary não permitiam predizer a resposta ao TI.

No entanto, esse achado é consistente com a literatura.

Sabe-se que o reflexo induzido pelo TI pode não ser idêntico aos ataques espontâneos, sendo as bradiarritmias as mais frequentes.^6^ A forma cardioinibitória, por vezes sem pródromos e com trauma significativo, é frequentemente registrada em pacientes submetidos a monitor de eventos implantável.^6^

Outro ponto importante a ser discutido é a utilização do acesso IV e sua possível interferência na análise da variabilidade RR e a alta incidência da necessidade de manobras farmacológicas para estabilização clínica dos pacientes.

A utilização do acesso IV seria uma “peça” inadequada que interfere na análise fisiopatológica da síncope vasovagal?

A síncope vasovagal é uma reação complexa e, além da idade, grande parte da variação de resposta entre os indivíduos também pode ser provocada por estímulos nocivos e estar relacionada a métodos de estudo como a punção venosa.^7 , 8^

Stevens, em 1966 demonstrou que a instrumentação e sua ansiedade associada aumentavam consideravelmente a probabilidade de uma reação hipotensiva. Essa observação foi consistente com estudos humanos anteriores mostrando vasodilatação nos músculos esqueléticos durante o estresse emocional.^3^

O acesso IV também apresentava a desvantagem de possível dor concomitante, poluindo a atmosfera autonômica do paciente.^9 , 10^

Entre os poucos estudos na população pediátrica que foram publicados para investigar a atividade dos barorreceptores e a variabilidade RR durante o TI, a maioria não usou acesso IV ou meios farmacológicos para evitar reações cruzadas aos receptores e alterações resultantes na fisiologia normal do reflexo barorreceptor.^10 - 12^

Fato que chama a atenção no estudo de Oliveira et al.^4^ é que entre os 54 pacientes com TI positivo, 9 (16,6%) precisaram de intervenção médica para estabilização clínica.^4^ Embora seja um resultado superior ao observado em outros estudos, os autores não analisaram esse subgrupo de pacientes.

A segurança do TI em crianças é provavelmente a maior preocupação. A maioria dos eventos adversos do TI em pacientes com coração estruturalmente normal está relacionada ao teste sensibilizado com isoproterenol.^13^ Em geral, os eventos mais significativos estão presentes em pacientes com forma cardioinibitória, mas a maioria desses pacientes se recuperou completamente após deitar-se.^13^

A hipotensão induzida pelo TI geralmente é autolimitada e se recupera 1 minuto após a inclinação para trás. No entanto, Wieling et al. relataram sete casos adultos com hipotensão prolongada pós-síncope (HPPS).^14^ Apesar do uso de nitrato sublingual nesses casos, o autor argumenta que o mecanismo de HPPS demonstrou ser a recuperação retardada de VS e CO devido à diminuição da contratilidade cardíaca, e não à vasodilatação mediada pela retirada simpática vascular.^15^

Os sinais e sintomas prodrômicos são mais frequentemente experimentados em indivíduos jovens quando a síncope vasovagal espontânea secundária à emoção de estresse medo-dor é iminente. Isso indica um controle autonômico mais robusto.^16^

Durante a HPPS, o barorreflexo arterial está com a função deprimida e a FC e a PA persistentemente baixas e inapropriadas são consistentes com a supressão sustentada dos mecanismos excitatórios. Esses pacientes podem ser incapazes de ativar as vias simpáticas centrais para superar a atividade vagal exagerada.^15^

Nossa hipótese é que esse aspecto esteja envolvido no número significativo de HPPS relatados neste artigo.

É relatada maior ativação simpática na posição supina no sexo masculino, no grupo com TI positivo, ao contrário da ausência de diferença entre os sexos durante a ortostase.Os autores enfatizam a diferença temporal entre a puberdade e o sexos, o que pode afetar as diferentes respostas autonômicas observadas.^4^

Seria possível acrescentar mais duas variáveis que poderiam contribuir para esse achado: o índice de massa corporal (IMC) e, principalmente, a altura dos pacientes.

O IMC é um marcador preciso de aumento do risco cardiovascular, mas também está relacionado à intolerância ortostática. O IMC de pacientes com síndrome de taquicardia ortostática postural com baixo volume sanguíneo foi significativamente menor do que o de pacientes com volume sanguíneo normal. Isso sugere que o IMC está relacionado ao volume sanguíneo.^17^

Por outro lado, a síncope vasovagal ocorre mais comumente em indivíduos mais altos, o que se justifica pela importância da altura (h) no estresse gravitacional.^2^

A direção do campo gravitacional da Terra perto da superfície é vertical, e a distância r é igual à altura do sistema: Ugr=gz x h. Isso explica porque o efeito da gravidade é significativo apenas na postura ortostática. Na postura horizontal, a altura do corpo humano, h, é muito pequena, tornando Ugr insignificante, mas aumenta muito durante a ortostase.^2^

Recentemente, Elias Neto et al. demonstraram que a velocidade de onda de pulso (VOP) de indivíduos jovens após a ortostase apresentou valores semelhantes aos encontrados em idosos na posição dorsal, corroborando o papel do aumento da VOP com o aumento do componente retrógrado da onda de pulso na adaptação do indivíduo à posição ortostática.^18^

Como o comprimento aórtico está relacionado com a altura corporal, a onda de reflexão arterial ocorre mais tardiamente em indivíduos mais altos. Não seria incongruente supor que, em indivíduos jovens, principalmente do sexo feminino, aqueles que apresentam níveis basais de pressão arterial mais baixos, a presença de uma aorta mais alongada poderia interferir no retorno do componente refletido às porções aórticas proximais de forma otimizada, o que poderia resultar na ativação de reflexos relacionados à gênese da síncope (por exemplo, o reflexo de Bezold-Jarisch).^18^

Assim, é possível considerar que a análise da influência da complacência aórtica sobre o barorreflexo pode permitir maior elucidação da adaptação à ortostase e da fisiopatologia da síncope neuromediada.

Vários mecanismos reguladores ocorrem instantaneamente em resposta aos efeitos da gravidade. O sistema regulador neural medeia o ajuste inicial à ortostase. Entretanto, a incorporação de parâmetros físicos e dinâmicos vasculares, particularmente das grandes artérias, provavelmente decorrentes da evolução da espécie humana frente ao estresse gravitacional, pode contribuir com peças importantes para o melhor entendimento da fisiopatologia da síncope vasovagal, particularmente na população jovem que se encontra em transição hormonal/estrutural adaptativa.
