# Supplementary material for: Que Peças Faltam no Quebra-Cabeça da Adaptação Cardiovascular ao Ortostatismo?
Source: Arq Bras Cardiol. 2023 Jul 27;120(7):e20230417. [Article in Portuguese] doi: 10.36660/abc.20230417 (PMC10382136; doi:10.36660/abc.20230417)
Supplement: Translation

# Translation: What Pieces are Missing in the Puzzle of Cardiovascular Adaptation to Orthostatism?

**Authors:** Jorge Elias

**Affiliations:** 1 Brasil Vitória Apart Hospital – Serviço de Eletrofisiologia, Serra, ES – Brasil; 2 Brasil Hospital Universitário Cassiano Antonio Moraes (Hucam) – Ufes, Vitória, ES – Brasil

**Keywords:** Syncope, Head-up Tilt Test, R-R Variability, Pulse Wave Velocity, Ortostasis

The bipedalism of the human species is an evolutionary milestone and has motivated extensive research in the physiological and anthropological areas.^1 , 2^

Although the gravitational force (+Gz), acting at a mean velocity of 9.8 m/s^2^ , creates a gradient of pressure at the level of the circulatory system, the human being is capable of maintaining an upright posture because the gravitational pressure is partially neutralized by autonomic and cardiovascular compensatory mechanisms that prevent hypotension and loss of consciousness would ensue after orthostasis.^1 - 3^

In this edition, Oliveira et al.^4^ contribute with a few more pieces to this complex adaptation puzzle to gravitational stress in children and adolescents.^4^

Recently, a decrease in the use of head-up tilt test (HUTT) in the investigation of syncope has been observed. Some authors have even argued that HUTT for the workup of syncope should be abolished due to the possibility that it fails to establish an explicit cause of syncope, is plagued by false positives, and never plays a role in guiding treatment.^5^

Even though the HUTT over time has seen its use in diagnostic stratification more related to specific subgroups of patients with a history of syncope, it persists as a safe, low-cost, reproducible, and low-risk diagnostic method for investigating the response to gravitational stress and to study the pathophysiology related to syncope and other pathologies related to maladjustment to orthostatic stress.

This observation is corroborated by the present study, where an integrated clinical approach, using the clinical history, the Calgary score, and the HUTT, made it possible to make the etiological diagnosis in the vast majority of patients, thus avoiding unnecessary tests.^4^

Some aspects stand out in this study.

The first was that several clinical variables and the Calgary scores did not allow for predicting the response to the HUTT.

Nevertheless, this finding is consistent with the literature.

It is known that Tilt-induced reflex may not be identical to spontaneous attacks, with bradyarrhythmias being the more frequent.^6^ The cardioinhibitory form, sometimes without prodromes and with significant trauma, is frequently recorded in patients undergoing implantable loop recorder.^6^

Another important point to discuss is the use of I.v. access and its possible interference in the analysis of RR variability and the high incidence of the need for pharmacological maneuvers for the clinical stabilization of patients.

Would using I.v. access be an inadequate “piece” that interferes with the pathophysiological analysis of vasovagal syncope?

Vasovagal syncope is a complex reaction, and, in addition to age, much of the variation between individuals may also be provoked by noxious stimuli and relate to study methods such as venipuncture.^7 , 8^

Stevens, in 1966, demonstrated that instrumentation and its associated anxiety increased considerably the likelihood of a hypotensive reaction. This observation was consistent with earlier human studies showing vasodilation in skeletal muscles during emotional stress.^3^

The I.v. access also had the disadvantage of possible accompanying pain, polluting the autonomic atmosphere of the patient.^9 , 10^

Among the few studies in the pediatric population that have been published to investigate the baroreceptor activity and R-R variability during HUTT, most did not use I.v. access or pharmacologic means to avoid cross-reactions to receptors and resulting changes in the normal baroreceptor reflex physiology.^10 - 12^

A fact that draws attention in the study by Oliveira et al.^4^ is that among the 54 patients with positive HUTT, 9 (16.6%) required medical intervention for clinical stabilization.^4^ Although it is a result superior to that observed in other studies, the authors did not analyze this subgroup of patients.

The safety of HUTT in children is probably of the greatest concern. The majority of adverse events of HUTT for patients with normal heart structure are reportedly related to the isoproterenol challenge.^13^ In general, the most significant events are present in patients with cardioinhibitory form, but most of these patients completely recovered after lying down.^13^

Hypotension induced by HUTT is usually self-limiting and recovers 1 minute after tilting back. However, Wieling et al. reported seven adult cases with prolonged post-faint hypotension (PPFH).^14^ Despite the use of sublingual nitrate in these cases, the author argues that the mechanism for PPFH has been demonstrated to be delayed recovery of SV and CO because of decreased cardiac contractility, not vasodilatation mediated by vascular sympathetic withdrawal.^15^

The prodromal signs and symptoms are more often experienced in young subjects when spontaneous vasovagal syncope secondary to fear-pain stress emotion is imminent. This indicates a more robust autonomic control.^16^

During PPFH, the arterial baroreflex is unloaded, and the persistent inappropriately low HR and BP are consistent with sustained suppression of excitatory mechanisms. These patients may be unable to activate the central sympathetic pathways to overcome exaggerated vagal activity.^15^

We hypothesize that this aspect is involved in the significant number of PPFH reported in this article.

Greater sympathetic activation in the supine position in males, in the group with positive HUTT, as opposed to the absence of difference between the sexes during orthostasis, is reported. The authors properly emphasize the temporal difference between puberty and genders, which could impact the different observed autonomic responses.^4^

It would be possible to add two more variables that could contribute to this finding: the body mass index (BMI) and, particularly, the height of the patients.

BMI is an accurate marker of increased cardiovascular risk but is also related to orthostatic intolerance. The BMI of patients with postural orthostatic tachycardia syndrome with a low blood volume was significantly lower than that of patients with a normal blood volume. This suggests that BMI is related to blood volume.^17^

On the other hand, vasovagal syncope occurs more commonly with taller individuals, which is justified by the importance of height (h) in gravitational stress.^2^

The direction of the Earth’s gravitational field close to the surface is vertical, and the distance r is equal to the system height: Ugr=gz x h. That explains why the effect of gravity is significant only in the orthostatic posture. In the horizontal posture, the height of the human body, h, is too little, making Ugr negligible, but it greatly increases during orthostasis.^2^

Recently, Elias Neto et al. demonstrated that the pulse wave velocity of young individuals after orthostasis showed values similar to those found in older people in the dorsal position, corroborating the role of increased pulse wave velocity with an increased retrograde wave in the individual’s adaptation to the orthostatic position.^18^

As the aortic length is related to body height, the arterial reflection wave occurs later in taller individuals. It would not be incongruous to suppose that, in young individuals, especially females, those who present lower basal levels of arterial pressure, the presence of a more elongated aorta could interfere with the return of the reflected component to the proximal aortic portions in an optimized way, which could result in the activation of reflexes related to the genesis of the syncope (for instance, the Bezold-Jarisch reflex).^18^

Thus, it is possible to consider that analyzing the influence of aortic compliance on the baroreflex can allow further elucidation of the adaptation to orthostasis and the physiopathology of neuromediated syncope.

Several regulatory mechanisms occur instantly in response to the effects of gravity. The neural regulatory system mediates the initial adjustment to orthostasis. However, the incorporation of physical and dynamic vascular parameters, particularly of the great arteries, probably arising from the evolution of the human species in the face of gravitational stress, may contribute with important pieces to a better understanding of the pathophysiology of vasovagal syncope and orthostatic hypotension, particularly in the young population who are in an adaptive hormonal/structural transition.
